# Assessment of an integrated therapeutic protocol for sheep with acute ruminal impaction: diagnostic and prognostic significance of rumen functions and hepatorenal biomarkers

**DOI:** 10.3389/fvets.2025.1587098

**Published:** 2025-05-22

**Authors:** Enas Elmeligy, Ebtsam S. Abdel-lah, Abdulaziz H. Almuhanna, Eman A. R. Abdelghffar, Mustafa Shukry, Mahmoud Saber, Ashraf M. Abu-Seida, Sayed Fathi El-Hawari, Laila A. Al-Shuraym, Lamya Ahmed Alkeridis, Khaled A. Khesruf, Arafat Khalphallah

**Affiliations:** ^1^Veterinary Teaching Hospital, Faculty of Veterinary Medicine, Assiut University, Assiut, Egypt; ^2^Department of Pharmacology, Faculty of Veterinary Medicine, Assiut University, Assiut, Egypt; ^3^Department of Clinical Sciences, College of Veterinary Medicine, King Faisal University, Al-Ahsa, Saudi Arabia; ^4^Department of Biology, College of Science, Taibah University, Yanbu, Saudi Arabia; ^5^Department of Zoology, Faculty of Science, Ain Shams University, Abbasseya, Egypt; ^6^Department of Physiology, Faculty of Veterinary Medicine, Kafrelsheikh University, Kafrelsheikh, Egypt; ^7^Division of Internal Medicine, Department of Medicine and Infectious Disease, Faculty of Veterinary Medicine, Cairo University, Giza, Egypt; ^8^Department of Surgery, Anesthesiology and Radiology, Faculty of Veterinary Medicine, Cairo University, Giza, Egypt; ^9^Department of Surgery, Anesthesiology, and Radiology, Faculty of Veterinary Medicine, Sohag University, Sohag, Egypt; ^10^Department of Biology, College of Science, Princess Nourah Bint Abdulrahman University, Riyadh, Saudi Arabia; ^11^Department of Animal diseases, Faculty of Veterinary Medicine, Aleppo University, Aleppo, Syria; ^12^Division of Internal Medicine, Department of Animal Medicine, Faculty of Veterinary Medicine, Assiut University, Assiut, Egypt

**Keywords:** acute ruminal impaction, electrolytes imbalance, hepatorenal functions, Osimi sheep, therapeutic regimen, rumen functions indices

## Abstract

**Introduction:**

Acute ruminal impaction is a metabolic disorder of the digestive system that happens in ruminants that have been fed a high amount of cereal grains, negatively impacting animal health and productivity. The present study clarified the diagnostic and prognostic significance of certain clinico-biochemical findings in evaluation of the efficacy of the applied therapeutic regimen in sheep with acute carbohydrate engorgement. This was conducted through monitoring changes in clinical findings, blood pictures, ruminal function biomarkers, serum hepatorenal indicators, and serum electrolytes indices in fattening Osimi sheep on days 0 pre-therapy and days 3, 7, 15, 30, 60, and 90 post-therapy.

**Methods:**

The study comprised fattening non-pregnant Osimi sheep (*n* = 100) with acute ruminal impaction. They were exposed to a 5-day integrated treatment regimen. It included IV infusion of sodium bicarbonate and glucose, an oral drenching of each of magnesium hydroxide, antibloat preparation (Bloatryal), and laxative powder (Apilax Powder). This was followed by injectable doses of flunixin meglumine, diphenhydramine HCl, clanobutin sodium, broad spectrum antibiotic (Combikel 20 + 20), and doramectin (a broad-spectrum anthelmintic). The investigated sheep had a full clinical examination and hemato-biochemical tests from day 0 (pre-therapy) up to day’s 3–90 post-therapy.

**Results and conclusion:**

The applied therapeutic regimen (1–5 days’ therapeutic program) was highly effective in cases of acute ruminal impaction in sheep, as evidenced by a clear improvement in their clinical health status (up to 15 days post-therapy) as well as restoring their reference intervals of ruminal functions biomarkers, blood picture indices, and hepatorenal functions throughout the current study (15–60 days post-therapy). Except for hepatorenal functions, all estimated laboratory indices restored their physiological intervals on the 15th day post-therapy. A 30- to 60-day follow-up period was required post-therapy until hepatorenal function restored their physiological reference intervals.

## 1 Introduction

Ruminal impaction is the most severe type of microbial fermentative illnesses associated with the rumen, and in rare cases, it is fatal in less than 24 h (1–3). Acute ruminal impaction in sheep with indigestible feed particles was recorded in a semi-arid region of Nigeria (1) and was clinically characterized by reduced appetite, symmetrical abdominal distension, emaciation, copious salivation, empty rectum, and recumbency (2, 3).

Many articles have described ruminal overload, and thus, its pathogenesis is more commonly discussed than that of the other stomach disorders in ruminants (4–6).

The illness spread among domestic ruminants, and substantial fatality rates were reported. Hazardous dyspepsia in goats results from the rapid intake of very hazardous quantities of highly fermentable carbohydrates-rich diets, mostly cereal grains. The morbidity rate in animals with ruminal impaction varies between 10 and 50%, whereas case fatality in untreated instances may approach 90%, compared to 30–40% in treated patients (2, 7–11).

Acute ruminal impaction is characterized by fast fermentation and the production of lactic acid as a result of excessive ingestion of highly fermentable carbohydrates, which is accompanied by a reduction in ruminal pH to physiologically inappropriate values (8, 12). This happens when animals consume an excess of concentrate feeds, e.g., if animals are suddenly fed without prior adaptation to such types of feeds; if animals suddenly consume large quantities of such feeds due to accidental access; or if animals are off feed then return to feed and have unrestricted access to concentrates (8–10). This disease condition in ruminants is more prevalent in group feeding than in solo or separate feeding; this may be attributed to social feeding behavior such as competitive intake, which encourage them to over consume (11–13). These feeds often contain cereal grains frequently employed in high-production diets, as well as fruit and root crops (sugar beets, feed beets, and potatoes). Starch and soluble carbohydrates cause an overgrowth of microorganisms that create glucose and organic acids. The produced acid stimulates and increases ruminal acidity (pH) and osmolality, kills other ruminal microorganisms, and causes forestomach or abomasum dysfunction and metabolic abnormalities (12–15).

Certain feedstuff properties lead to the acidity of the rumen fluid. Cereal grains often have less buffering than fibrous forages. The less fiber content and small forage particle size of cereal grains result in less salivation at the time of ingestion and less rumination later; hence, the salivary buffering system decreases when concentrate grain feeds are eaten or digested. Some silages are rich in carbohydrates and lactic acid; therefore, more lactic acid is formed after ingestion and fermentation (4, 8, 10, 11).

The release of lactic acid induces chemical irritation of the rumen wall (rumenitis), and its absorption results in lactic or toxic acidosis. The pathophysiologic effects are hemoconcentration, cardiovascular failure or collapse, renal insufficiency or failure, muscle weakness, shock, and death. The survived animals may develop mycotic rumenitis in a few days, liver necrobacillosis many weeks or months later, or chronic laminitis, and evidence of ruminal scared is found at slaughters (9). The thinning of rumeno-reticular contents, which turn porridge-like and odorous, indicates the presence of fermentation (4). The cornified epithelium lining the wall of these organs is readily detached, showing hemorrhagic surfaces. Abomasitis, fungal infections, and rumenitis may follow the acute episode of this disease.

Researchers have extensively researched acute ruminal impaction (9–11, 15), but accurate diagnosis and appropriate rapid treatment remain the only means to preserve precious animals. As a result, the current study clarified the diagnostic and prognostic significance of certain clinico-biochemical findings in evaluation of the efficacy of a certain therapeutic regimen in sheep with acute carbohydrate engorgement through monitoring changes in clinical findings, blood pictures, ruminal function biomarkers, serum hepatorenal indicators, and serum electrolytes indices in fattening Osimi sheep on days 0 pre-therapy and days 3, 7, 15, 30, 60, and 90 following treatment.

## 2 Materials and methods

### 2.1 Animals

The study comprised 100 non-pregnant fattening Osimi sheep ranging in age from 8 to 12 months (10.33 ± 1.55). Their body weight varied from 40 to 50 kg (45.27 ± 3.42). They belonged to private farms in Egypt at Assiut, Cairo, Qena, Sohag, and Kafrelsheikh governorates. All tested sheep had an acute ruminal impaction as a result of consuming significant amounts of easily fermentable carbohydrate-rich feed, such as cereal grains widely used in high-production diets, bread, maize, corn, and/or root crops (e.g., potatoes). The diseased ewes were subjected to an integrated therapeutic regimen for the acute ruminal impaction that lasted 5 days. The diseased ewes were subjected to a thorough clinical examination with evaluations of hematological parameters, blood pH, ruminal function metabolism, serum electrolytes, and hepatorenal indicators on days 0 (pre-therapy), 3, 7, 15, 30, 60, and 90 post-therapies.

### 2.2 Samples

Blood and ruminal fluid samples were collected from the examined sheep on day 0 (pre-therapy), as well as on days 3, 7, 15, 30, 60, and 90 after treatment. Blood samples were divided into two parts: whole blood samples collected in vacutainer tubes with ethylenediamine tetra acetic acid (EDTA) and kept at 4°C, and blood serum samples taken in plain vacutainer tubes and maintained at −20°C until analysis. All precautions were taken during blood sample collection and preparation as previously stated (16, 17) since they were obtained from the jugular vein after sanitizing the region of the jugular furrow.

The rumen juice samples were collected shortly before morning feeding (17–19). By using a rubber stomach tube, 50 mL of rumen fluid or juice was taken from each sheep throughout the previously mentioned sampling days.

### 2.3 Therapeutic strategy

The sick sheep with acute ruminal acidosis was subjected to therapeutic regimen according to Radostits et al. (2), Smith (3), Garry and McConnel (5), Constable et al. (6), Navarre et al. (19), Enemark (20), and Karapinar et al. (21). The treatment lasted for successive 5 days until the diseased animals restored their healthy status. The integrated regimen included slow IV infusion of sodium bicarbonate 5% (ADWIA Co., Cairo, Egypt) for 30 min at a dose of 500 mL/sheep every 24 h for 5 days. Then, a 5-day IV infusion of glucose (Glucose 5% w/v B.P 2013^®^, ADWIA Co., Cairo, Egypt) was administered at a dosage of 500 mL per sheep every 24 h. Oral drenching of magnesium hydroxide (Drench Prod sachets^®^, Macon, France) was given as an antacid and a moderate laxative at a dose of 100 gram mixed with 1.5 L warm water q 12 h for 5 days (Macon, France). Flunixin meglumine (Flunixin Injection^®^, ANADA 200–308, Norbrook Laboratories Ltd., Newry, Co. Down, N. Ireland) was also given slowly intravenous at a dosage of 2 mL/45 kg bwt every 24 h for 5 days. Diphenhydramine HCl (Histacure, Pharma Swede, Cairo, Egypt) was given as an antihistaminic at a dosage of 0.5–1.1 mg/1 kg body weight (bwt) every 24 h via SC route for 5 days. Antibloat preparation containing dimethicone (25 mg), turpentine oil (0.3 mL), and anise oil (0.01 mL) (Bloatryal^®^, Pharma Swede, Cairo, Egypt) was given as an oral dosage of 50 mL q24 h for 5 days. Each animal got an oral dose of apilax powder (Laxavet Plus^®^, Pharma Swede-Egypt), with each pack containing magnesium carbonate (87 g), sodium carbonate (10 g), and gentiana herbal (2 g). Apilax powder was administered for 5 days in the form of 50 gm dissolved in half a liter of fresh water/animal q 12 h for the first day and then every 24 h for the next 4 days. For 5 consecutive days, all affected sheep received 5 mL clanobutin sodium/sheep q24h (Bykahepar^®^, Schering-Plough Animal Health; MSD Animal Health, Kenilworth, New Jersey, USA). The first dosage of Bykahepar^®^ was administered via IV route, while the subsequent four doses were administered via the S/C route. Doramectin (Dectomax^®^10 mg/mL injectable solution, Zoetis Co., USA) was administered as a broad-spectrum anthelmintic medication to gastrointestinal worms at a dose of 1 mL/50 kg by S/C injection on day 0 and repeated on day 14. The diseased sheep were given procaine benzylpenicillin and dihydrostreptomycin sulfate (Combikel 20 + 20^®^, KELA N.V., Hoogstraten, Belgium) as a broad-spectrum antibiotic at a dose of 5 mL/50 kg bwt every 24 h for 5 days via the SC route.

### 2.4 Clinical examination

All examined animals had a thorough clinical evaluation. The examination included an assessment of the abdomen, posture and gait, symptoms of dehydration, and the mucous membrane. Furthermore, temperature, pulse rates, respiration rates, capillary refill time (CRT), and ruminal motility were calculated. The appetite was scored using Jackson and Cockcroft (22), and Nagy and Pugh (23) methods. The appetite scores ranged from 0, 1, 2, and 3 for anorexia, severely reduced appetite, moderately reduced appetite, and normal appetite, respectively.

### 2.5 Rumen function indicators

Rumen pH (SMP1 pH meter) was determined immediately after collecting ruminal fluids, followed by sample sieving. The sieved samples were split and kept for the measurement of total protozoa count (TPC) as stated by Dehority (24), ammonia concentration as consistent with Conway (25) and Zapletal (26), and total volatile fatty acid (TVFA) concentration as mentioned by Warner (27), and Cottyne and Boucque (28). Centrifuged rumen liquor supernatant was deep-frozen at −20°C until tested. The sample for TVFAs and ammonia estimation was divided into two parts: the primary portion (2 mL) for determining ammonia nitrogen values after preservation by adding liquid paraffin, and the second portion (2 mL) for TVFA levels after preservation by adding phosphoric acid (2 mL) and 1 mL acid N/10, according to Warner (27).

To measure TPC, a well-mixed ruminal fluid sample was promptly fixed in an equivalent volume of formalin (18.5%) (24). For TPC, a solution of methyl green formalin saline (MFS) was used to stain and store an aliquot of each juice sample (29, 30). MFS was employed as a nuclear stain, and Lugol’s iodine was used to visualize skeletal plates (31, 32). A Neubauer hemocytometer counting chamber was used to determine protozoal concentrations. This counting chamber had narrow grooves cut at regular intervals. This formula calculated the number of cells per 1 mL of rumen contents: *N* = 10/4 × a × d (*N* = number of ruminal ciliates per 1 mL of ruminal contents, a = number of ciliates in 4 divisions of the Neubauer hemocytometer, d: sample dilution) (30, 32, 33).

### 2.6 The venous blood pH and blood picture indices

Following the collection of whole blood samples, blood pH was determined immediately using an SMP1 pH meter. The red blood corpuscle count (RBCs), total leukocytic count (TLC), hemoglobin concentration (Hb), and packed cell volume (PCV) were measured using standard manual methods (16, 34, 35).

### 2.7 Serum electrolyte indices

The Spectro Ultraviolet-Vis RS spectrophotometer (Labomed, Inc., Los Angeles, CA, USA) was used to assess serum levels of all serum electrolytes using commercially supplied kits. Blood electrolyte values, including sodium, potassium, calcium, and phosphorus, were estimated using commercial kits obtained from Bio Diagnostic Company (Egypt), while serum chloride was determined using Spinreact reagent kits (Spinreact Company, Spain).

### 2.8 Serum renal functions and hepatic biomarkers

The Spectro Ultraviolet-Vis RS spectrophotometer (Labomed, Inc., Los Angeles, CA, USA) was used to measure blood renal function indicators and hepatic biomarkers using commercially available kits.

Renal function indicators such as blood urea nitrogen (BUN) and creatinine were tested with kits and reagents purchased from Egypt’s Bio Diagnostics Company. Gamma Trade Company reagent kits were used to examine hepatic function by measuring blood aspartate aminotransferase (AST) activity, serum alkaline phosphatase (ALP) activity, and serum glucose concentrations.

### 2.9 Statistical analysis

The data were analyzed with the SPSS statistical software program for Windows (ver. 16.0; SPSS, USA). The normal distribution of all parameters was tested using Kolmogorov–Smirnov normality test. All parameters were normally distributed. The data obtained from the clinical findings and laboratory analyses were analyzed using general linear model repeated measures ANOVA, with a significance level at a *p*-value of < 0.05. The significance of differences was assessed on specified sample days, namely, days 0, 3, 7, 15, 30, 60, and 90.

## 3 Results

### 3.1 Clinical findings

The affected sheep (*n* = 100) with acute ruminal impaction had classic signs at day 0 before beginning the therapy program, including anorexia (*n* = 100), ataxia (*n* = 100), evidence of dehydration (*n* = 100), and substantial left abdominal distension (*n* = 100). Dehydration symptoms included injected mucous membranes, chilly clammy skin (lasting more than 30 s), dullness and depression, prolonged CRT, and tough tight skin with failure to retract selected skin folds. Abnormal lung and heart sounds, and enlarged lymph nodes were not detected. A clear improvement in the general health status of affected ewes was observed as a result of the therapeutic regimen, with some of them beginning to restore recover their baseline health values on the third day following therapy; however, complete disappearance of anorexia, ataxia, left distended abdomen, and dehydration signs was reported on the fifteenth day following therapy initiation (Table 1).

**Table 1:** Common clinical findings in sheep (*n* = 100) with acute ruminal impaction.

Follow-up period	Anorexia	Posture and gait (ataxia)	Prominent LAD	Signs of dehydration				
				Injected MMs	Picking up a skin fold (fell immediately back)	CRT (<2 s) Constable et al. (6)	Depression and dullness	A cold clammy skin (>30 s) Jackson and Cockcroft (22)
Day 0	100 (100)*	80 (80)	100 (100)	100 (100)	0 (0)	0 (0)	80 (80)	88 (88)
Day 3	80 (80)	72 (72)	60 (60)	72 (72)	12 (12)	40 (40)	60 (60)	48 (48)
Day 7	24 (24)	12 (12)	12 (12)	32 (32)	48 (48)	76 (76)	40 (40)	32 (32)
Day 15	0 (0)	0 (0)	0 (0)	0 (0)	100 (100)	100 (100)	0 (0)	0 (0)
Day 30	0 (0)	0 (0)	0 (0)	0 (0)	100 (100)	100 (100)	0 (0)	0 (0)
Day 60	0 (0)	0 (0)	0 (0)	0 (0)	100 (100)	100 (100)	0 (0)	0 (0)
Day 90	0 (0)	0 (0)	0 (0)	0 (0)	100 (100)	100 (100)	0 (0)	0 (0)

*Number of sheep (%). LAD, left abdominal distension; CRT, capillary refill time; MMs, mucous membranes.

**Figure 1:**
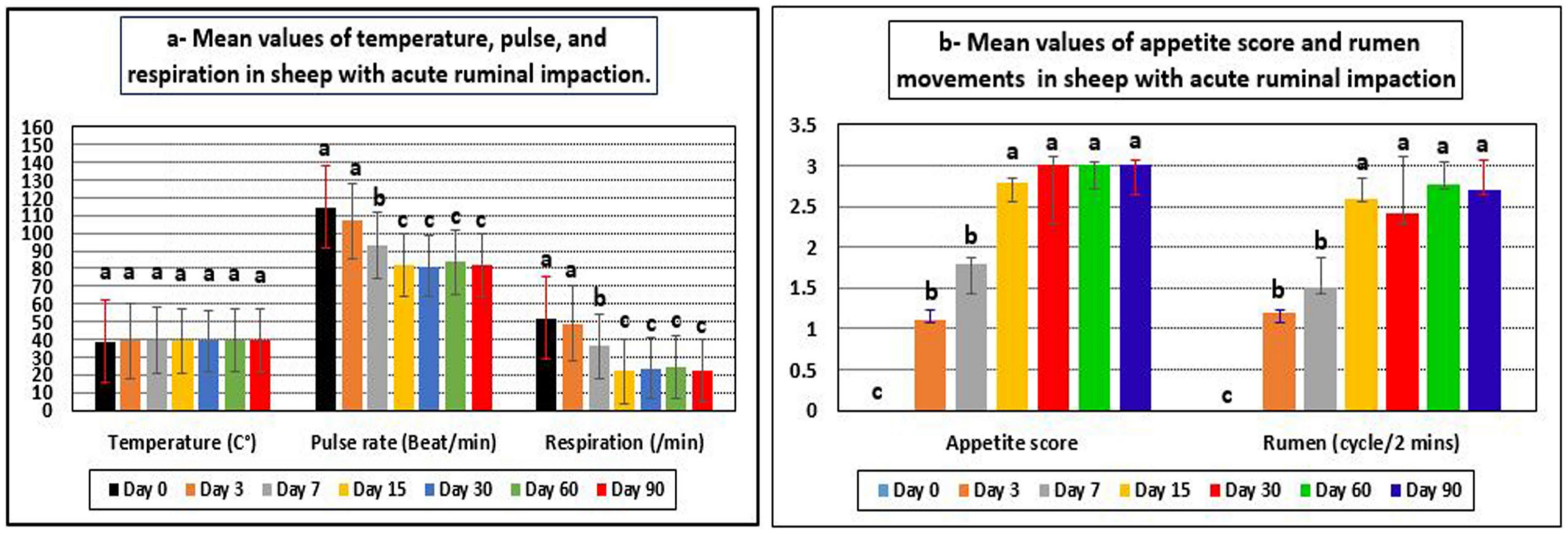
Mean values of temperature, pulse, respiration **(a)**, appetite score, and rumen movements **(b)** in sheep with acute ruminal impaction. ^abc^Means above each bar with different superscript letters between different sampling times, i.e., days 0, 3, 7, 15, 30, 60, and 90, were significantly different (*p* < 0.05).

Appetite score, pulse rates, respiration rates, and rumen movement all revealed significant changes during the current investigation, although temperature readings showed no significant changes and remained within their reference range (Table 2 and Figure 1). Before treatment, the tested sheep had significantly lower appetite scores and rumen movements (*p* < 0.05), while pulse rates and respiratory rates were significantly higher (*p* < 0.05) compared to their post-therapy values, especially at days 15–90. However, all parameters returned to their reference intervals after 15–60 days of treatment. Pulse rates, respiration rates, and ruminal motility all improved significantly following treatment, particularly 15 days later. There were no recorded fatality rates among the diseased sheep.

**Table 2:** Mean values (M ± SD) of appetite score, temperature, pulse, respiration, and rumen movements in sheep (*n* = 100) with acute ruminal impaction.

Follow-up period	Appetite score	Temperature (C^°^) (38.5–40) Jackson and Cockcroft (22)	Pulse rate (Beat/min) (70–90) Radostits et al. (2), Jackson and Cockcroft (22)	Respiration (/min) (20–30) Jackson and Cockcroft (22)	Rumen (cycle/2 min) (2–4) Igbokwea et al. (1)
Day 0^#^	0^c^	38.82 ± 0.51^a^	114.67 ± 6.08 ^a^	52.06 ± 6.72^a^	0^c^
Day 3	1.10 ± 0.35^b^	39.21 ± 0.34^a^	107.22 ± 8.36 ^a^	48.73 ± 5.84^a^	1.20 ± 0.38^b^
Day 7	1.80 ± 0.31^b^	39.52 ± 0.60^a^	93.08 ± 5.11^b^	36.04 ± 4.58^b^	1.5 ± 0.68^b^
Day 15	2.80 ± 0.41^a^	39.15 ± 0.71^a^	82.25 ± 2.44^c^	22.01 ± 2.55^c^	2.60 ± 0.34^a^
Day 30	3^a^	39.52 ± 0.62^a^	81.10 ± 4.09^c^	23.44 ± 4.76^c^	2.42 ± 0.41^a^
Day 60	3^a^	39.55 ± 0.44^a^	83.61 ± 5.13^c^	24.03 ± 2.98^c^	2.77 ± 0.28^a^
Day 90	3^a^	39.37 ± 0.36^a^	82.09 ± 4.36^c^	22.48 ± 2.53^c^	2.71 ± 0.35^a^

^#^Treatment day. ^abc^Means within the same column with different superscript letters between different sampling times, i.e., days 0, 3, 7, 15, 30, 60, and 90, were significantly different (*p* < 0.05).

### 3.2 Therapeutic regimen trials

The current study used an integrated therapeutic regimen for acute ruminal impaction in sheep based on a multi-drug regimen continued up to 5 days that included slow IV infusion of sodium bicarbonate 5% followed by IV infusion of glucose with oral drenching of magnesium hydroxide and slow IV injection of Flunixin meglumine. Diphenhydramine HCl 20 mg was used as antihistaminic and oral antibloat preparations [25 mg dimethicone, 0.3 mL turpentine oil, and 0.01 mL anise Oil] commercially named Bloatryal. Each animal received oral dose of laxative powder [magnesium carbonate (87 g), sodium carbonate (10 g), and gentiana herbal (2 g)] named Apilax Powder, i.e., Laxavet Plus followed by injectable doses of clanobutin sodium [Bykahepar^®^]. Doramectin was also used as prophylactic doses of a broad spectrum anthelmintic drug for GI. The sick sheep received procaine benzylpenicillin and dihydrostreptomycin sulfate (Combikel 20 + 20) as a broad spectrum antibiotic. The therapeutic regimen was highly effective in treating acute ruminal impaction in sheep. A clear improvement was noticed in the general health status of affected ewes. The improvement included complete disappearance of dehydration signs and observable improvement in ruminal metabolism biomarkers. Correction of dehydration, acid–base imbalance, and electrolyte disturbances were achieved at the 15th day following the start of therapy (Tables 1–5). Furthermore, hepatorenal function required up to 30–60 days of surveillance after treatment began to restore their physiological condition (Tables 6, 7).

### 3.3 Ruminal function indicators

**Figure 2:**
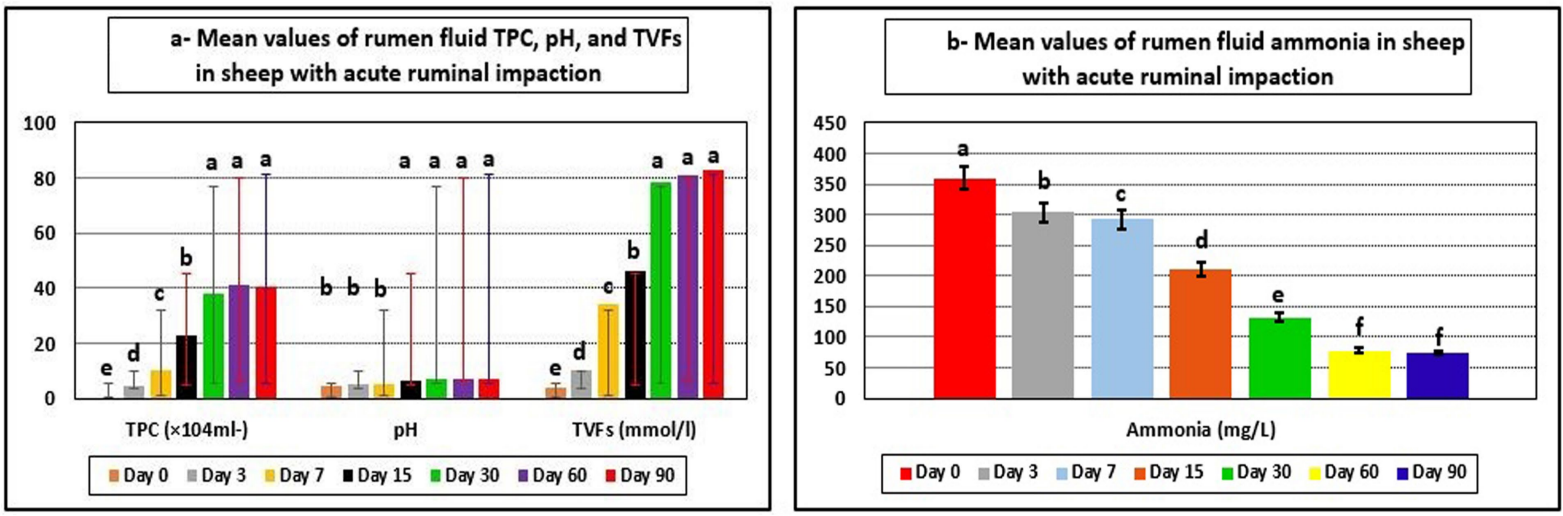
Mean values of rumen fluid TPC, pH, TPC **(a)**, and ammonia **(b)** in sheep with acute ruminal impaction. ^a-f^Means above each bar with different superscript letters between different sampling times, i.e., days 0, 3, 7, 15, 30, 60, and 90, were significantly different (*p* < 0.05). TPC, total protozoal count; TVFAs, total volatile fatty acids.

Ruminal function markers showed significant changes. TPC, rumen juice pH, and TVFAs in the affected ewes on day 0 (pre-therapy) were all lower than their reference ranges. Furthermore, rumen ammonia concentrations exceeded their reference ranges. The integrated therapy improved ruminal function biomarkers in diseased ewes, while TPC, rumen juice pH, and TVFAs were significantly elevated (*p* < 0.05). As a result, ruminal ammonia concentrations were significantly reduced (*p* < 0.05) at post-therapeutic days, particularly 15–90 days, compared to day 0. The ruminal fluid levels of TPC, rumen juice pH, TVFAs, and ammonia concentrations approached their reference limits by the 30th day following therapy initiation (Table 3 and Figure 2).

**Table 3:** Mean values (M ± SD) of rumen fluid parameters in sheep (*n* = 100) with acute ruminal impaction.

Follow-up period	TPC (×10^4^mL^−^) (28.13 ± 4.13) Baraka (49)	pH (6.4–6.8) Jasmin et al. (50)	TVFAs (mmol/L) (70–150) McDonald et al. (51)	Ammonia (mg/L) (50–300) McDonald et al. (51) (40–240) Satter and Slyter (53)
Day 0^#^	0^e^	4.61 ± 0.13^b^	4 ± 0.21^e^	360 ± 8.10^a^
Day 3	4.37 ± 2.16^d^	4.83 ± 0.04^b^	10.17 ± 2.01^d^	304 ± 10.88^b^
Day 7	10.03 ± 1.20^c^	5.00 ± 0.73^b^	34.17 ± 6.01^c^	292 ± 7.01^c^
Day 15	22.73 ± 2.64^b^	6.41 ± 0.17^a^	46.22 ± 5.35^b^	211 ± 5.35^d^
Day 30	38.26 ± 4.37^a^	6.74 ± 0.16^a^	78.44 ± 3.98^a^	132 ± 8.79^e^
Day 60	41.12 ± 6.71^a^	6.82 ± 0.36^a^	80.65 ± 4.54^a^	78 ± 3.22^f^
Day 90	40.77 ± 5.92^a^	6.91 ± 0.31^a^	82.75 ± 5.27^a^	74 ± 2.81^f^

TPC, total protozoal count; TVFAs, total volatile fatty acids. ^#^Treatment day. ^a-f^Means within the same column with different superscript letters between different sampling times, i.e., days 0, 3, 7, 15, 30, 60, and 90, were significantly different (*p* < 0.05).

### 3.4 Complete blood picture indices and venous blood pH

**Figure 3:**
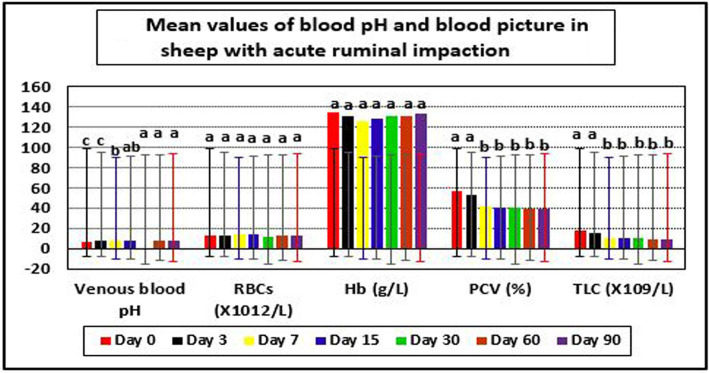
Mean values of blood pH and blood picture in sheep with acute ruminal impaction. ^abc^Means above each bar with different superscript letters between different sampling times, i.e., days 0, 3, 7, 15, 30, 60, and 90, were significantly different (*p* < 0.05). RBCs, red blood corpuscles; Hb, hemoglobin; PCV, packed cell volume; TLC, total leukocytic count.

In diseased ewes at day 0, the current study reported significant changes in some blood picture indices such as PCV and TLC, even though RBCs and Hb values were within their reference intervals. PCV and TLC values were above their reference values indicting dehydration and leukocytosis, respectively. The same results were reported for venous blood pH, but their values were below their reference ranges indicating metabolic acidosis (Table 4 and Figure 3).

**Table 4:** Mean values (M ± SD) of blood pH and blood picture in sheep (*n* = 100) with acute ruminal impaction.

Follow-up period	Venous blood pH (7.32–7.54) Smith (63)	RBCs (_X_10^12^/L) (8–18) Jackson and Cockcroft (22), Radostits et al. (64) or (6.2–15.5) Aitken (65)	Hb (g/L) (90–150) Jackson and Cockcroft (22), Radostits et al. (64)	PCV (%) (27–45) Jackson and Cockcroft (22), Radostits et al. (64)	TLC (_X_10^9^/L) (4–12) Jackson and Cockcroft (22), Radostits et al. (64)
Day 0^#^	7.03 ± 0.11^c^	12.38 ± 2.08^a^	134.44 ± 3.95^a^	56.32 ± 4.06^a^	17.88 ± 5.20^a^
Day 3	7.11 ± 0.10^c^	12.88 ± 2.83^a^	130.65 ± 4.05^a^	53.26 ± 2.86^a^	15.22 ± 3.17^a^
Day 7	7.26 ± 0.06^b^	13.77 ± 2.12^a^	126.01 ± 2.84^a^	42.05 ± 4.38^b^	10.06 ± 1.38^b^
Day 15	7.35 ± 0.04^ab^	13.38 ± 2.01^a^	128.22 ± 5.11^a^	40.22 ± 5.11^b^	10.57 ± 2.05^b^
Day 30	7.43 ± 0.05^a^	12.06 ± 2.33^a^	131.17 ± 3.98^a^	39.92 ± 3.62^b^	9.97 ± 2.94^b^
Day 60	7.44 ± 0.04^a^	13.25 ± 2.78^a^	130.57 ± 3.92^a^	38.74 ± 4.07^b^	9.58 ± 2.19^b^
Day 90	7.41 ± 0.03^a^	13.07 ± 1.88^a^	134.00 ± 3.77^a^	38.82 ± 4.26^b^	9.36 ± 2.74^b^

^#^Treatment day. RBCs, red blood corpuscles; Hb, hemoglobin; PCV, packed cell volume; TLC, total leukocytic count. ^#^Treatment day. ^abc^Means within the same column with different superscript letters between different sampling times, i.e., days 0, 3, 7, 15, 30, 60, and 90, were significantly different (*p* < 0.05).

The applied therapy substantially raised venous blood pH values (*p* < 0.05) at day’s 15–90 post-therapy, compared to day 0. PCV and TLC levels were significantly lower on days 15–90 compared to day 0. Following therapy, the affected sheep regained physiological levels for venous blood pH and PCV at day 15, as well as TLC on day 7. At day 15 post-therapy, metabolic acidosis with decreased blood pH and dehydration with higher PCV levels were corrected in recovered sheep (Table 4 and Figure 3).

### 3.5 Serum electrolytes

**Figure 4:**
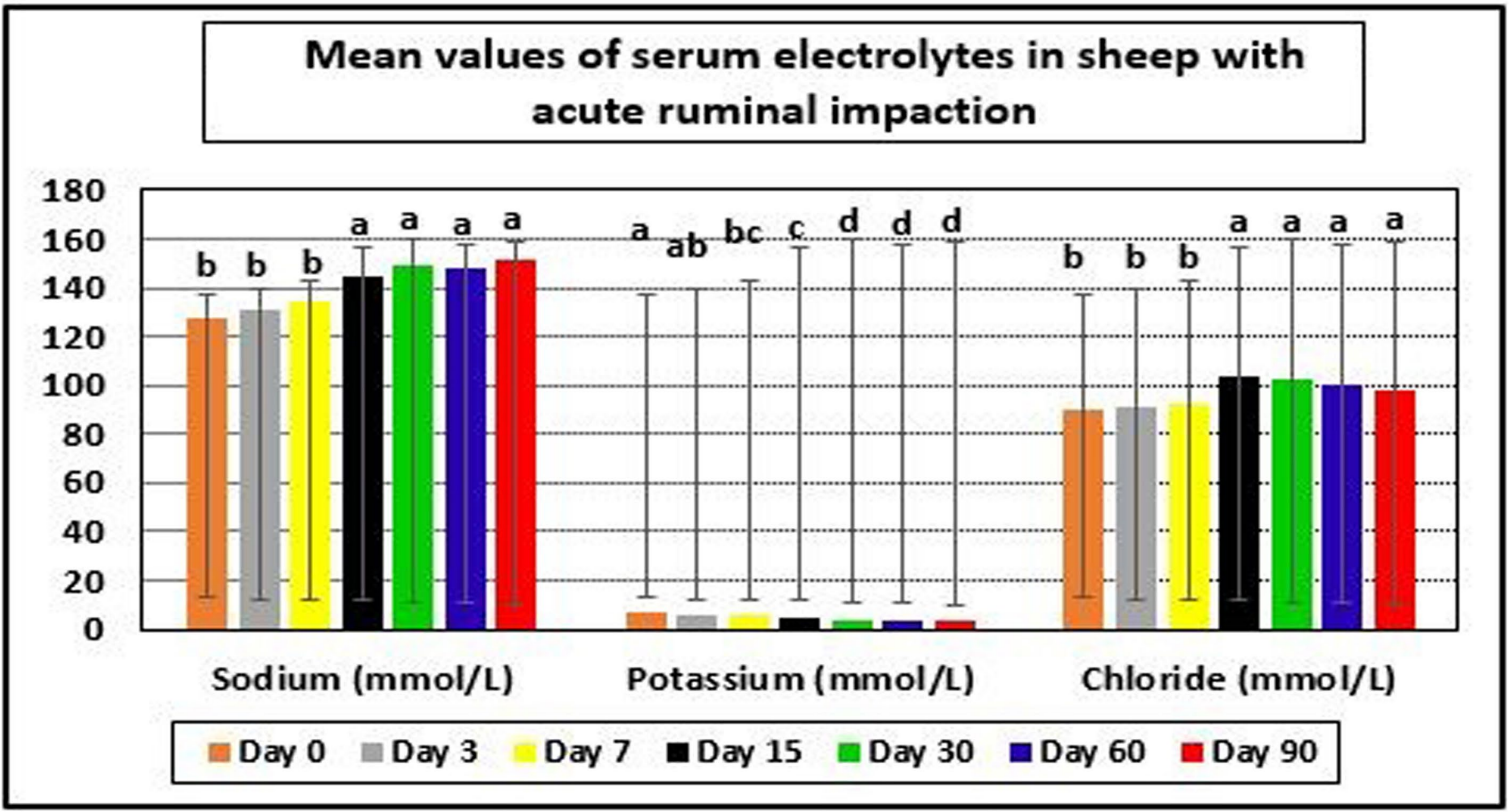
Mean values of serum electrolytes in sheep with acute ruminal impaction. ^a-d^Means above each bar with different superscript letters between different sampling times, i.e., days 0, 3, 7, 15, 30, 60, and 90, were significantly different (*p* < 0.05).

**Figure 5:**
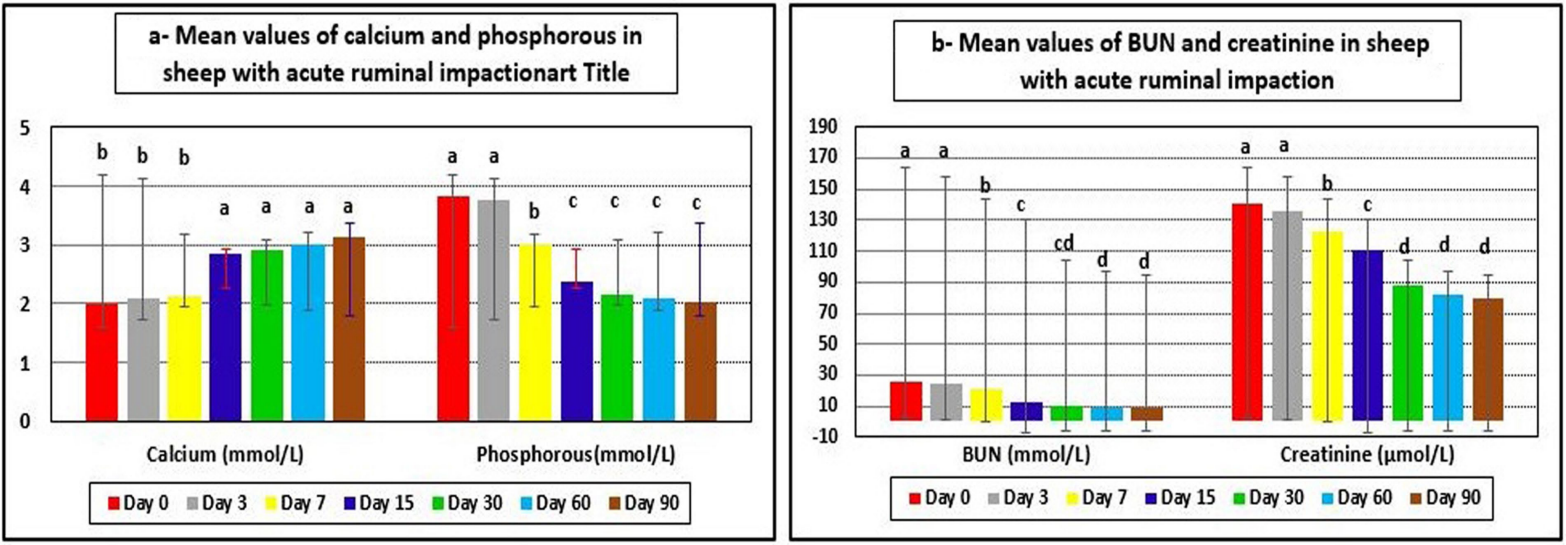
Mean values of calcium, phosphorous, BUN, and creatinine in sheep with acute ruminal impaction. ^a-d^Means above each bar with different superscript letters between different sampling times, i.e., days 0, 3, 7, 15, 30, 60, and 90, were significantly different (*p* < 0.05). BUN, blood urea nitrogen.

The examined ewes had hyponatremia, hypochloremia, hypocalcemia, hyperkalemia, and hyperphosphatemia on day 0 before starting the treatment plan. At days 15–90 post-therapy, serum salt, chloride, and calcium levels were substantially higher (*p* < 0.05), whereas potassium and phosphate levels were significantly lower (*p* < 0.05) in ill sheep compared to pretreatment values on day 0. Electrolyte balance was restored, and blood values of electrolytes such as sodium, potassium, chloride, calcium, and phosphates approached their reference ranges by the 15th day after therapy (Tables 5, 6 and Figures 4, 5a).

**Table 5:** Mean values (M ± SD) of serum electrolytes in sheep (*n* = 100) with acute ruminal impaction.

Follow-up period	Sodium (mmol/L) (145–152) Jackson and Cockcroft (22), Radostits et al. (64)	Potassium (mmol/L) (3.9–5.4) Jackson and Cockcroft (22), Radostits et al. (64), Aitken (65)	Chloride (mmol/L) (95–103) Jackson and Cockcroft (22), Radostits et al. (64)
Day 0^#^	127.95 ± 5.96^b^	7.02 ± 1.44^a^	90.06 ± 6.73^b^
Day 3	131.25 ± 6.03^b^	6.28 ± 0.92^ab^	91.15 ± 4.47^b^
Day 7	134.18 ± 6.39^b^	5.87 ± 1.28^bc^	92.45 ± 6.05^b^
Day 15	144.71 ± 4.58^a^	5.01 ± 1.70^c^	103.35 ± 5.13^a^
Day 30	149.59 ± 4.40^a^	4.06 ± 0.87^d^	102.45 ± 3.87^a^
Day 60	148.06 ± 4.63^a^	3.91 ± 0.91^d^	100.36 ± 4.11^a^
Day 90	151.29 ± 3.73^a^	4.07 ± 0.77^d^	97.49 ± 3.81^a^

^#^Treatment day. ^a-d^Means within the same column with different superscript letters between different sampling times, i.e., days 0, 3, 7, 15, 30, 60, and 90, were significantly different (*p* < 0.05).

**Table 6:** Mean values (M ± SD) of calcium, phosphorous, BUN, and creatinine in sheep (*n* = 100) with acute ruminal impaction.

Follow-up period	Calcium (mmol/L) (2.88–3.20) Jackson and Cockcroft (22), Radostits et al. (64)	Phosphorous (mmol/L) (1.62–2.36) Jackson and Cockcroft (22), Radostits et al. (64)	BUN (mmol/L) (3.5–12.5) Jackson and Cockcroft (22), Radostits et al. (64)	Creatinine (μmol/L) (70–105) Jackson and Cockcroft (22), Radostits et al. (64)
Day 0^#^	1.98 ± 0.37^b^	3.82 ± 0.1^a^	26.08 ± 4.66^a^	140.07 ± 12.44^a^
Day 3	2.08 ± 0.21^b^	3.77 ± 0.09^a^	24.00 ± 2.15^a^	135.33 ± 9.06^a^
Day 7	2.12 ± 0.45^b^	3.00 ± 0.21^b^	20.58 ± 4.01^b^	122.33 ± 7.97^b^
Day 15	2.84 ± 0.14^a^	2.36 ± 0.16^c^	13.06 ± 3.97^c^	110.49 ± 8.85^c^
Day 30	2.92 ± 0.09^a^	2.15 ± 0.23^c^	10.36 ± 1.78^cd^	87.62 ± 4.58^d^
Day 60	3.02 ± 0.1^a^	2.08 ± 0.09^c^	9.55 ± 2.66^d^	82.11 ± 6.07^d^
Day 90	3.13 ± 0.22^a^	2.01 ± 0.11^c^	9.02 ± 2.28^d^	79.68 ± 5.13^d^

^#^Treatment day. BUN, blood urea nitrogen. ^#^Treatment day. ^a-d^Means within the same column with different superscript letters between different sampling times, i.e., days 0, 3, 7, 15, 30, 60, and 90, were significantly different (*p* < 0.05).

### 3.6 Renal functions and hepatic biomarkers

**Figure 6:**
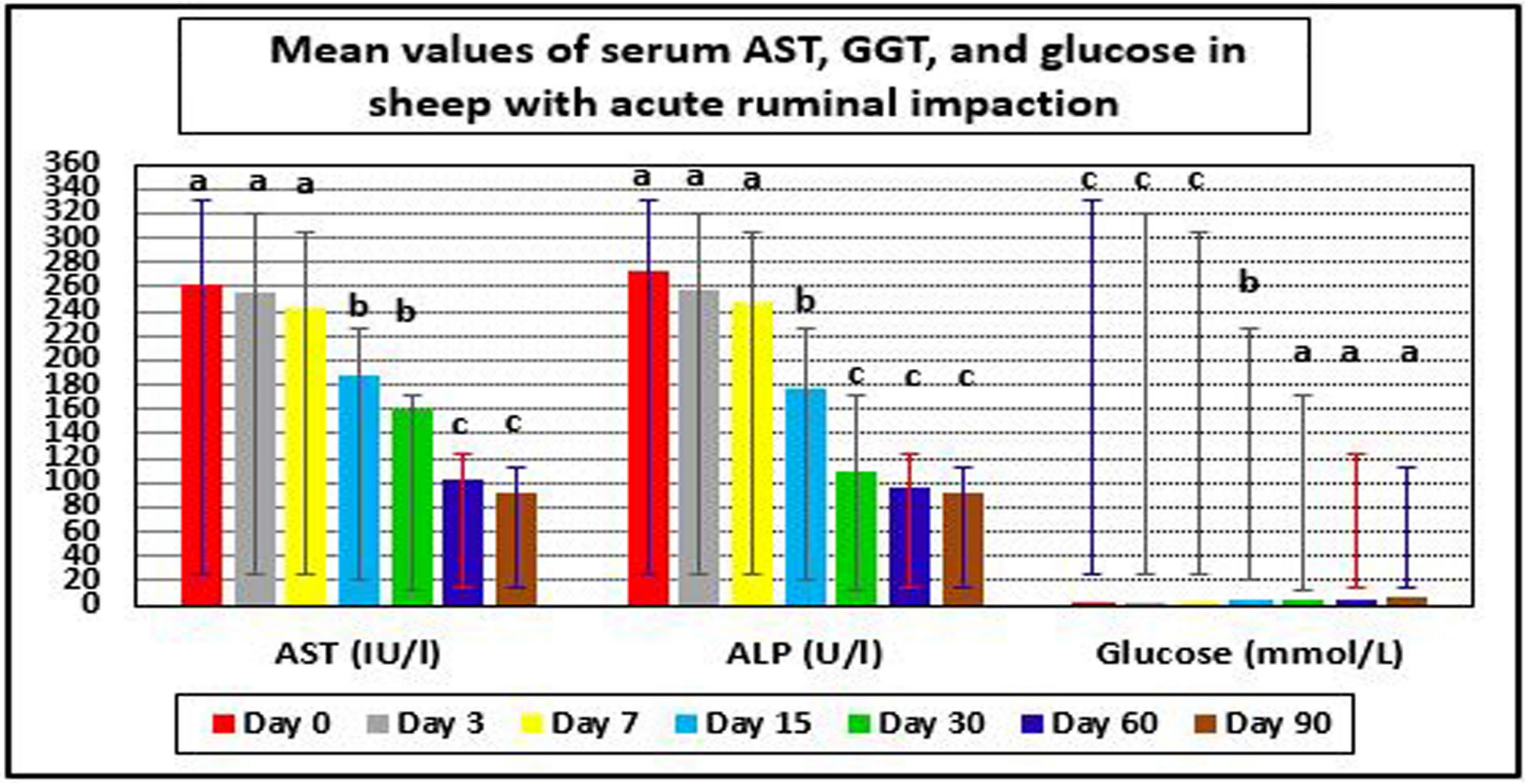
Mean values of serum AST, GGT, and glucose in sheep with acute ruminal impaction. ^a-d^Means above each bar with different superscript letters between different sampling times, i.e., days 0, 3, 7, 15, 30, 60, and 90, were significantly different (*p* < 0.05). AST, aspartate aminotransferase; ALP, alkaline phosphatase.

Hepatorenal function examination revealed substantial changes in the examined sheep. Renal biomarkers such as BUN and serum creatinine levels as well as hepatic function indicators such as AST, ALP, and glucose exhibited obvious changes. At day 0 prior to therapy, the kidney biomarkers raised above their reference values. Serum concentrations of BUN and creatinine were considerably (*p* < 0.05) lower following treatment, especially on days 7–90, compared to pre-therapy levels on day 0. On day 30, serum BUN and creatinine levels reached their reference ranges in recovered sheep, indicating that renal function had improved. Hepatic function indictors included serum AST, ALP, and glucose, which showed significant changes in diseased sheep with acute ruminal impaction. Their serum activities of AST and ALP were significantly (*p* < 0.05) dropped, while those of glucose were significantly (p < 0.05) increased at days 15–90 following therapy comparing with their values at day 0. By the 30th–60th day, hepatic function indicators had returned to their normal range, indicating that hepatic function had improved (Table 7 and Figures 5b, 6).

**Table 7:** Mean values (M ± SD) of serum AST, GGT, and glucose in sheep (*n* = 100) with acute ruminal impaction.

Follow-up period	AST (IU/L) (60–280) Jackson and Cockcroft (22), Radostits et al. (64) (32–97) Aitken (65)	ALP (U/L) (70–390) Jackson and Cockcroft (22), Radostits et al. (64) (128.67 ± 12.65) Novoselec et al. (79)	Glucose (mmol/L) (1.7–3.6) Jackson and Cockcroft (22), Radostits et al. (64)
Day 0^#^	262.14 ± 22.06^a^	272.18 ± 36.88^a^	2.82 ± 0.09^c^
Day 3	256.33 ± 17.44^a^	258.22 ± 43.05^a^	3.06 ± 0.2^c^
Day 7	242.12 ± 20.15^a^	247.01 ± 30.15^a^	3.20 ± 0.1^c^
Day 15	188.64 ± 22.16^b^	176.02 ± 20.68^b^	4.20 ± 0.22^b^
Day 30	160.15 ± 18.36^b^	110.11 ± 19.37^c^	5.48 ± 0.09^a^
Day 60	103.02 ± 10.24^c^	97.22 ± 8.61^c^	5.22 ± 0.16^a^
Day 90	92.55 ± 8.06^c^	91.22 ± 6.14^c^	5.83 ± 0.33^a^

AST, aspartate aminotransferase; ALP, alkaline phosphatase. ^#^Treatment day. ^a-d^Means within the same column with different superscript letters between different sampling times, i.e., days 0, 3, 7, 15, 30, 60, and 90, were significantly different (*p* < 0.05).

## 4 Discussion

### 4.1 Clinical findings

Ruminal overload in domestic ruminants was well recognized as a managemental disease for many years. The disease was clinically characterized by loss of appetite, abdominal distension, diarrhea, weakness, depression, dullness, and inactivity. It was commonly reported due to accidental ingestion of large amounts of cereal grains or their flour kept for human consumption (4, 6, 10, 19). With agreement with Gumbrell (4), Constable et al. (6), and Navarre et al. (19), the present study revealed that the affected sheep with ruminal overload showed characteristic signs at day 0 before applying the therapeutic program which included anorexia, disturbances in posture and gait mainly ataxia, clear signs of dehydrations, and prominent left abdominal distension. Moreover, the current study reported a clear improvement of the general health status of affected ewes as a result of therapeutic regimen whereas some of ewes started to restore their physiological status at the 3rd following therapy where complete disappearance of these clinical findings was reported at the 15th days following therapy. On the other side, the previous articles added that the severity of ruminal acidosis and its signs varied considerably, depending on the amount and type of ingested carbohydrate-rich feed and the degree of previous acclimatization of ruminal microbes to the consumed carbohydrate substrates. The disease could range from a mild form of indigestion (Simple form) to an overwhelming toxemia that might be difficult to differentiate from other acute toxicities or various endotoxemic diseases (5, 6).

Due to the current study appetite score, heart rates, respiratory rates, and rumen movement showed remarkable changes throughout the study; meanwhile, temperature values showed no remarkable changes whereas they were within their reference values mentioned by Radostits et al. (2) and Jackson and Cockcroft (22). These results contradicted with Mohamed Nour et al. (10) and Ullah et al. (11). Furthermore, Ullah et al. (11) reported subnormal rectal temperature before treatment in all the lactic acidotic goats as compared to control goat group. After treatment, the elevation in body temperature was a positive indication toward the recovery. They also added that the decreased rectal temperature noted in their study might be attributed to lactic acidosis, leading to dehydration. Karapinar et al. (21) reported that the body temperature initially was elevated but might decrease as the condition worsened or the animal became toxic. On the other side, the current study mentioned clear improvements in appetite score, pulse rates, respiratory rates, and ruminal motility in diseased sheep with acute ruminal impaction post-therapy particularly at days 15–60 comparing with their day zero values (pre-treatment). Appetite score, pulse rates, respiratory rates, and ruminal motility restored their reference intervals stated by Radostits et al. (2) and Jackson and Cockcroft (22) at days 15–60. Appetite score and rumen movements were significantly dropped, while pulse rates and respiratory rates were significantly elevated in sheep with acute ruminal impaction at day 0 comparing with their values post-therapy. Other literature reported polypnea and tachycardia in lactic acidosis in small ruminants (2, 9, 11, 36, 37) that might be attributed to stimulation of respiratory center by elevating carbon-dioxide (CO2) tension of blood and reduced blood pH (11) as well as dehydration and electrolyte imbalance status associated with acute ruminal acidosis led to reduced blood volume and caused hemoconcentration which in turn induced myocardial contractility for pumping blood and elevating pulse rates (6). Furthermore, clinical manifestations with lactic acid poisoning in sheep and goat varied with the type and amount of feed ingested and the time elapsed following ingestion. The clinical signs appeared in the first 12 to 36 h after ingestion or intake of the offending feed where they varied from anorexia, depression, weakness, and reluctant to move to recumbency in an animal having severe circulatory shock due to lactic acidosis. Dehydration signs oftenly were severe, and evidence of toxemic signs was reported, i.e., injected mucous membranes and highly injected scleral capillaries. Colic, rumen stasis, bilateral ventral abdominal distention, and a “splashy” feel to the rumen also might be observed. Diarrhea and dehydration might develop (19, 36, 38, 39). The diarrheal output ranged from paste-like feces to very watery with foam, sometimes with pieces of clearly observable grains. Dehydration status, lactic acidosis, and toxemia might result in ataxia, seizures, head pressing, opisthotonos, and other neurologic abnormalities (4, 19, 40). Furthermore, several toxic factors had been involved in acute ruminal acidosis in addition to lactic acid (13, 15). The altered metabolism of the ruminal population microflora had been reported to generate great quantities of histamine, methanol, ethanol, tyramine, and tryptamine. These might play an important role in the pathogenesis of ruminal impaction, but conclusive evidence was lacking. Histamine had been indicated as an agent in the development of laminitis which sometimes accompanied ruminal acidosis. However, histamine was imperfectly absorbed from the rumen, especially at very low pH levels. The destruction of ruminal gram negative bacteria had been suggested to release large quantities of endotoxin for absorption through damaged mucosal surfaces. Endotoxin could contribute to most of the clinical findings associated with rumen overload such as ruminal atony, poor tissue perfusion with cardiovascular deterioration, weakness, and depression. Increased ruminal and blood concentrations of endotoxin and increased blood arachidonic acid metabolites had been found in cattle with experimentally induced ruminal acidosis, but their importance in naturally occurring disease was not clear (5, 41–44).

### 4.2 Therapeutic regimen trials

Treatment of acute carbohydrate engorgement of ruminants was aimed at correcting cardiovascular shock, dehydration, acidosis, and toxemia and removing or neutralizing the offending feedstuffs. Intravenous fluids containing 5% sodium bicarbonate should be administered (6, 38, 45). The current study used an integrated therapeutic regimen for acute carbohydrate engorgement in sheep depended upon the use of group of medicaments lasted for successive 5 days and included slow IV infusion of sodium bicarbonate 5% followed by IV infusion of glucose with oral drenching of magnesium hydroxide. Other previous reports added that calcium might be indicated and could be added to the intravenous fluids (as calcium gluconate). It was not recommended for clinician to mix sodium bicarbonate and calcium salts (3, 5, 19). Ullah et al. (11) supported the therapeutic use magnesium hydroxide in lactic small ruminants with acute ruminal acidosis as they said that on the basis of change in ruminal and blood pH, protozoa motility test, and recovery rate of animals, it was found that magnesium hydroxide was more effective than sodium bicarbonate as it caused greater increase in ruminal pH and improved general body condition and rapid recovery. The change in pH was significant after the administration of drugs. Magnesium hydroxide was indicated more than sodium bicarbonate in acute ruminal acidosis as reported by Aslan et al. (46), Smith and Correa (47), and Galip (48). Slow IV injection of Flunixin meglumine was used during the current study to relieve pain and as anti-endotoxic in sheep with ruminal overload. On the other hand, oral fluids were contraindicated because absorption was diminished, potentially increasing the rumen distention and worsening the animal’s discomfort. Non-steroidal anti-inflammatory drugs (NSAIDs) were indicated to control the pain and inflammation of toxemia (Flunixin meglumine, 1.1–2.2 mg/kg IV) (5, 19). The current study used in sheep with lactic acidosis diphenhydramine HCl 20 mg used as antihistaminic as well as oral antibloat preparations commercially named Bloatryal. Each animal received oral dose of laxative powder named Apilax Powder, i.e., Laxavet Plus followed by injectable doses of clanobutin sodium (Bykahepar^®^). Doramectin was also used as prophylactic doses of a broad spectrum anthelmintic drug for GI. The sick sheep received procaine benzylpenicillin and dihydrostreptomycin sulfate as broad spectrum antibiotic. This therapeutic program achieved great efficacy in dealing with lactic acid poisoning in sheep through a clear improvement of the general health status of affected ewes with complete disappearance of dehydration signs as well as observable improvement in ruminal metabolism biomarkers. Correction of dehydration, acid–base imbalance, and electrolytes disturbances were reported at the 15th days following the onset of therapy. Moreover, hepatorenal function needed up to 30–60 days of observation following treatment onset until restoring their physiological status back. The currently used therapeutic regimen in sheep with acute ruminal impaction was supported by several studies in which oral administration of magnesium hydroxide and magnesium oxide (1 g/kg) might neutralize the acidic pH and was sufficient in mild cases. However, if much of the feed still remained in the rumen, these two alkalizing agents would only work temporarily. Oral antibiotics were advisable to kill rumen microbes and stop fermentation. It was believed that these agents were contraindicated; however, the gram negative anaerobes that needed to flourish to reestablish normal rumen microflora were susceptible to most antimicrobials effective against *Lactobacillus* species. Removing the substrate for growth of *Lactobacillus* organisms was more effective. Because orogastric tubes with large enough bores for reflux of feedstuffs were too large for sheep and goats, rumenotomy was indicated in severe cases to remove the feed. After the rumen pH was corrected, transfaunation of the rumen microflora with approximately 1 qt of rumen fluid from another small ruminant was beneficial (21). The current study used also broad spectrum antibacterial was ac combination between Procaine benzylpenicillin and dihydrostreptomycin sulfate in sheep with acute ruminal impaction. On the other side, Navarre et al. (19) supported these results whereas they mentioned that bacterial leakage into the rumen wall, liver, and systemic circulation made antimicrobial therapy necessary. The systemic antimicrobial agent of choice was penicillin (procaine penicillin G, 22,000 IU/kg IM twice daily) because anaerobes were the most likely offending organisms. With aggressive treatment, the prognosis for short-term survival was good. Feed (grass hay only) and water should be restricted until return of rumen contractions, to prevent overdistention of the rumen.

### 4.3 Ruminal function indicators

Ruminal function indicators throughout the present study showed significant changes in diseased ewes with lactic acidosis at day 0 (pre-therapy), whereas values of TPC, rumen juice pH, and TVFAs were lower than their reference ranges reported by Khaled and Baraka (17), Navarre et al. (19), Baraka (49), Jasmin et al. (50), McDonald et al. (51), and Akbar et al. (52). Furthermore, values of rumen ammonia concentrations were above their reference intervals mentioned by McDonald et al. (51), Satter and Slyter (53), and Rianto et al. (54). The study reported significant improvement in ruminal function biomarkers due to therapy in diseased ewes whereas values of TPC, rumen juice pH, and TVFAs were remarkably elevated; hence, values of ruminal ammonia concentrations were significantly reduced at post-therapeutic days particularly 15–60 days comparing with their values at day 0 in ewes with lactic acidosis. Ruminal fluid values of TPC, rumen juice pH, TVFAs, and ammonia concentrations reached their reference ranges by the 30th day following therapy onset. These results were supported by Garry and McConnel (5), Mohamed Nour et al. (10), Ullah et al. (11), Navarre et al. (19), Haji Hajikolaei et al. (37), Aslan et al. (46), and Crichlow (55). Furthermore, Ullah et al. (11) reported that the reduction in pH of the ruminal fluid noted in small ruminants with lactic acidosis corresponded to increase production of volatile fatty acids such as acetic, propionic, and butyric acid. The decrease in pH of the rumen favored the growth of streptococci with decline in the number of normal Gram-negative bacteria and protozoa, which further aggravated the process of lactic acid production. Gumbrell (4) added that the pH of the rumen contents was usually less than 5 in cases of acute carbohydrate engorgement (the values increase after death), and microscopic examination will show only bacteria and dead protozoa. The other literature revealed that several factors appeared to affect the density and composition of the rumen ciliate protozoa. These factors related to the type and amount of feed consumed, frequency of feeding, rumen pH, and turnover rate (56–58). Moreover, Ullah et al. (11) stated ruminal protozoa were absent in the ruminal fluids collected from lactic acidotic animals, while a large number of protozoa were examined in the ruminal fluid obtained from healthy control animals. After 24 h of treatment, again the protozoa motility test was repeated for all the groups. A slight to moderate amount of protozoa were observed in the ruminal fluids collected from magnesium hydroxide-treated ruminant group and sodium bicarbonate-treated ruminant group. Protozoa were not observed in the ruminal fluids of untreated grain overload animals. Gumbrell (4) reported the same results in sheep as the pH of the rumen contents was usually less than 5 in cases of acute carbohydrate engorgement (the values increase after death), and microscopic examination will show only bacteria and dead protozoa. Navarre et al. (19) and Braun et al. (36) also added that the rumen fluid pH might fall below 5.5 in acute carbohydrate engorgement. The fluid itself was milky gray, and particles of the inciting feed might be noted. Protozoa usually were reduced in number or absent, and large gram-positive rods (*Lactobacillus* species) might be seen on Gram staining. In addition to the VFAs and rumen ciliate protozoa or TPC, the previous reports clarified the effects on the animal from the ruminal fluid changes were numerous and detrimental. In the early stages of the acidic fermentation, the VFAs were produced in abundance. Although VFA production decreases as the microbes were increasingly inhibited, and VFA concentrations remain elevated in advanced acidosis. The VFAs were much weaker acids than lactic acid; thus, as pH drops, they accepted hydrogen from lactic acid and serve as buffers in the fluid, so that a greater proportion of the VFAs existed in the non-dissociated state. This form was more readily absorbable than free ions through the ruminal wall. During absorption process, some VFAs undergone metabolism by the ruminal wall epithelium, leading to the release of lactate and ketone bodies into the circulation. Excessive absorption of the volatile fatty acids led to systemic acidosis, and circulating lactate and VFAs might also directly damage the liver. In addition, the high concentration of non-dissociated VFAs at the ruminal epithelium provided a strong inhibitory effect on reticuloruminal motility and led to ruminal stasis. This effect tended to protect the animal because it reduced the absorption of detrimental fermentation products from the rumen (59–62).

### 4.4 Complete blood picture indices and venous blood pH

Ullah et al. (11) reported that the blood pH of lactic acidotic group was found to be lower than the blood pH of healthy control group. The blood pH of treated lactic acidotic group either with magnesium hydroxide or with sodium bicarbonate was found to be higher than the blood pH of untreated lactic acidotic control group. It was also a diagnostic point for diseased condition. These results were in agreement with the current results which showed that with exception for RBCs and Hb values, the whole blood picture indices and venous blood pH showed significant alterations at day 0 in diseased sheep with acute ruminal impaction where they were either below their reference ranges mentioned by Smith (63) for venous blood pH indicating metabolic acidosis or above their reference values mentioned by Radostits et al. (64); Jackson and Cockcroft (22) for PCV and TLC indicating dehydration and leukocytosis, respectively. RBCs and Hb values were within their reference intervals reported by Jackson and Cockcroft (22), Radostits et al. (64), and Aitken (65). These findings were supported by Braun et al. (36), Cao et al. (66), and Patra et al. (67). With referring to the current study, the significant drop in blood pH in sheep with acute ruminal impaction pre-therapy comparing to post-treated cases might be owned to over-distention of rumen which impeded venous return to heart. This factor impaired hepatic perfusion and poorer lactic acid utilization which in turns led to systemic lactic acidosis, manifesting decreased blood pH (11). Accordingly, throughout the current study, values of venous blood pH were significantly increased; meanwhile, PCV and TLC values were remarkably decreased due to treatment particularly at days 15–60 post-therapy when they compared with their values at day 0 in sheep with lactic acidosis. The affected ewes, i.e., with acute ruminal impaction, restored their physiological values for venous blood pH and PCV at day 15 while for TLC at day 7 following treatment onset. The metabolic acidosis associated with lower blood pH and dehydration associated with elevated PCV values were corrected in recovered sheep at day 15 post-therapy. Navarre et al. (19) also added that laboratory data in acute carbohydrate engorgement in small ruminants were consistent with dehydration (increased PCV and total protein, prerenal azotemia) and metabolic acidosis (36). Furthermore, the leukogram varied in appearance, ranging from normal to a degenerative left shift, depending on the severity of the case (19). Surprisingly, there was at the most only a mild increase in the white blood cell count (68, 69), and endotoxin was rarely identified in the plasma of cattle and sheep with acute ruminal acidosis (69, 70). Furthermore, the degree of hemoconcentration, as referred by hematocrit, elevated with the amount of fluid withdrawn from the extracellular fluids space into the rumen and probably given the best single indicator of clinical severity in ruminal acidosis (6, 70). The hematocrit elevated from a value of approximately 34–50 to 60% in the terminal stages of acute ruminal acidosis in sheep and cattle and was accompanied by a fall in blood pressure (6, 68, 69).

### 4.5 Serum electrolytes

Blood pH, bicarbonate, and base excess fell markedly whereas plasma l-lactate and inorganic phosphate concentrations raised in ruminants with acute ruminal acidosis. In almost all cases, there was a mild hypocalcemia, which was probably caused by a temporary reduction in feed intake and gastrointestinal movements. Serum concentrations might drop to between 6 and 8 mg/dL (1.5–2.0 mmol/L) (6). Lactic acid was a strong corrosive agent that could destroy the ruminal epithelium, giving rise to the name *toxic rumenitis*. The increased ruminal fluid osmolality also damaged the epithelium as extracellular water influx across the epithelium occurred in response to osmotic pressure imbalance and disturbed sodium transport (14). The effects of epithelial destruction could be far-reaching because the damage persisted after resolution of the acute acidosis (7, 71). With referring to the present study, fattening sheep with acute carbohydrate engorgement showed observable changes in serum values of electrolytes at day 0 before applying the therapeutic plan whereas hyponatremia, hypochloremia, and hypocalcemia were reported. Serum concentrations of sodium, chlorides, and calcium were below their reference ranges mentioned by Jackson and Cockcroft (22), Radostits et al. (64), and Aitken (65). Moreover, hyperkalemia and hyperphosphatemia were reported in affected sheep at day 0 in which serum concentrations of potassium and phosphate were still above their reference intervals reported by Jackson and Cockcroft (22), Radostits et al. (64), and Aitken (65). The sick sheep with acute ruminal impaction at day 0 was suffering from systemic metabolic acidosis as well as clear dehydration due to drop in blood pH (below 5). These findings were confirmed by Smith (3) and Garry and McConnel (5). Furthermore, the osmotic pressure of the ruminal fluid increased as lactic acidosis developed. In a normal animal, ruminal osmolality was still relatively stable at approximately 280 mOsm/L, but osmolality might double in some cases of acute acidosis. Lactic acid accounted for a major fraction of the increase, but some of the components of this change remained unidentified. The increased osmolality inhibited and killed some of the microflora and drew fluid into the rumen, mostly from the extracellular compartment. This accounted for the increased ruminal fluid volume, ruminal distention, and severe dehydration observed clinically. The loss of circulating fluid volume led to circulatory impairment, decreased renal blood flow and glomerular filtration, and in some cases eventual anuria. Poor peripheral circulation resulted in hypoxic metabolism and contributed to systemic acidosis (3, 6, 7, 12, 13). Following the onset of the therapeutic regimen (Days 15–60 post-therapy), the affected sheep with ruminal acidosis showed significant elevations in serum levels of sodium, chloride, and calcium as well as significant drop in serum values of potassium and phosphate comparing with their zero day values pretreatment. Electrolyte balance was achieved, and hyponatremia, hyperkalemia, hypochloremia, hypocalcemia, and hyperphosphatemia were corrected whereas serum concentrations of sodium, potassium, chloride, calcium, and phosphates reached their reference intervals by the 15th days following therapy onset. On the other side, previous reports interpreted the metabolic acidosis and electrolyte imbalance whereas they revealed that although the systemic acidosis that developed with acute ruminal acidosis disease was owned to ruminal lactic acid absorption, such absorption did not appear to occur readily (13). Lactate was absorbed from the rumen at a much slower rate than were the volatile fatty acids because it was highly ionized at a pH near physiologic normal, which tended to inhibit absorption. At lower pH of ruminal fluid, the rumen became static, thereby also inhibiting absorption. The hypertonicity of the ruminal fluid further reduced absorption of lactate and other substances (14). It appeared that the peak entry of lactate into the circulation occurred in the early phases of the disease. Before the onset of complete stasis of intestine, some lactate might be absorbed from the intestinal tract from fluid passing through intestine. Many experimental trials did not show the development of severe systemic acidosis in the early phase of the disease. It might be that a large component of the later severe systemic acidosis was attributable to circulatory insufficiency rather than to absorption of lactic acid (5).

### 4.6 Renal functions and hepatic biomarkers

Hepatorenal function assessment showed dramatic changes in sheep with acute ruminal impaction as the renal biomarkers included BUN and serum creatinine levels as well as hepatic function indicators included AST, ALP, and glucose, which showed observable alterations. The renal biomarkers in affected sheep was above their reference intervals stated by Jackson and Cockcroft (22) and Radostits et al. (64) at day 0 before treatment. This might refer to moderate degree of renal insufficiency that might be owned to dehydration status and reduce renal blood flow that interpreted the higher serum values of BUN and creatinine in ewes with acute ruminal impaction at day 0 pretreatment. These results were supported by Smith (3) and Constable et al. (6). There was association between the reduction in both of rumen ammonia concentration and serum level of BUN where the two parameters were remarkably decreased (58). This could be explained as BUN concentrations were affected by ammonia absorption rates to the liver; Payne and Payne (72) added also that ammonia was absorbed to the liver for urea formation and the urea to blood for formation of BUN. Payne and Payne (72) mentioned that rumen ammonia was absorbed to the liver for formation of urea and the urea to blood for BUN formation. Both of rumen urea and ammonia were used by the rumen bacteria for the synthesis of bacterial protein, and bicarbonates in the rumen were required for the carbon skeleton formation to induce the conversion of simple nitrogenous compounds into more complex molecules of bacterial protein. Visek (73) mentioned that rumen urea concentrations were changed according to the rate of production and absorption of ammonia nitrogen, as well as on the rate of ammonia detoxification into urea in the liver. Moreover, the current study also mentioned that serum concentrations of BUN and creatinine were significantly reduced after treatment in sheep with lactic acidosis particularly at days 7–60 comparing with their values pre-therapy whereas they reached their reference intervals post-therapy at day 30 which might indicate improvement of renal function.

On the other side, ruminal acidosis was considered one of the primary causes of mycotic rumenitis and mycotic omasitis, although other predisposing caused chemical damage and might lead to abscess formation, diffuse cellulitis, or perforation and peritonitis. If the animal survived the acute acidosis, it might succumb to secondary ruminal damage (3, 74, 75). Alternatively, the rumen might heal uneventfully, leaving scars in the ruminal wall, but bacterial access to the circulation through these chemical lesions could result in hepatic abscessation, a common problem of animals fed high-concentrate rations (76, 77). These results confirmed the results of the current study where great improvements in hepatic function indicators, i.e., AST, ALP, and glucose, were demonstrated in ovine lactic acidosis cases post-therapy. Their serum concentrations and activities were significantly reduced for serum AST and ALP; hence, they were significantly increased for serum glucose at days 15–60 following therapy comparing with their values at day 0. The previous reports added that liver enzymes such as gamma-glutamyl transpeptidase (GGT), AST, and lactate dehydrogenase (LDH) might be elevated on serum biochemical analysis in ruminal overload (21, 38). Furthermore, with liver impairment or ruminal wall damage, toxin absorption and clearance were likely to be altered. Premature delivery and retained placenta might occur in pregnant animals after acute ruminal acidosis, possibly resulting from the effects of these circulatory toxins and metabolites (78). The present study reported that hepatic function biomarkers became within their reference interval mentioned by Jackson and Cockcroft (22), Radostits et al. (64), Aitken (65), and Novoselec et al. (79) and restored their physiological status by the 30th–60th days which might indicate improvement of hepatic functions.

## 5 Conclusion

The applied therapeutic regimen (1–5 days’ therapeutic program) was highly effective in cases of acute ruminal impaction in sheep, as evidenced by a clear improvement in their clinical health status (up to 15 days post-therapy) as well as restoring their reference intervals of ruminal functions biomarkers, blood picture indices, and hepatorenal functions throughout the current study (15–60 days post-therapy).

Except for hepatorenal functions, all estimated laboratory indices restored their physiological intervals, and correction of hyponatremia, hyperkalemia, hypochloremia, hypocalcemia, and hyperphosphatemia had been demonstrated on the 15th day after therapy.

A 30- to 60-day follow-up period was required post-therapy until hepatorenal function restored their physiological reference intervals.

Generally, the study had several limitations such as studying concurrent ruminant diseases and some epidemiological studies, where further future studies will be necessary to manage these limitations. Future study should address concurrent infections and broader epidemiological trends.

## Data Availability

The raw data supporting the conclusions of this article will be made available by the authors, without undue reservation.
